# Is Selfie Behavior Related to Psychological Well-being?

**DOI:** 10.11621/pir.2021.0302

**Published:** 2021-09-15

**Authors:** Elena A. Nikitina

**Affiliations:** a Institute of Psychology, Russian Academy of Sciences, Moscow, Russia

**Keywords:** selfie, psychological well-being, self-esteem, social support, motivation to success, motivation to avoid failure

## Abstract

**Background:**

The reasons and consequences of people’s activity on social networks have not been sufficiently studied. Most studies have focused on identifying the dangers and risks associated with posting self-portraits on social networks, but it is an open question as to whether such behavior serves to increase people’s psychological well-being.

**Objective:**

We asked ourselves what are the main motives for publishing selfies and whether online activity contributes to psychological well-being.

**Design:**

Our study involved 96 respondents from Moscow, mainly psycho logy students, who provided information about their activity on social networks, and completed questionnaires on their motivation, social support, and psychological well-being.

**Results:**

Three main motives for publishing selfies were identified: 1) to increase self-esteem; 2) maintain social contacts; and 3) preserve and exchange information. The higher the ratio of selfies taken to preserve information, the higher was the user’s psychological well-being. We found significant differences between the characteristics of those participants with high and low activity, and larger and smaller numbers of “friends” in social networks. For those with high activity, their sense of psychological well-being was largely determined by interaction with others. For these persons, communication (including with virtual contacts) was the important resource of well-being. The other group was less dependent on others, and for them, psychological well-being was not related to their activity on social networks.

**Conclusion:**

Our results confirm the connection between the personality traits and characteristics of the respondents’ network behavior and their psychological well-being. The study showed that the type of correlation constellation differs between groups of respondents depending upon how much or how little they are oriented toward social support.

## Introduction

The passion for taking and publishing selfies (photo self-portraits) which has spread in recent years, requires serious sociological, cultural, and, most importantly, psychological research. On November 19, 2013 the Oxford Dictionary officially announced that “selfie” was their Word of the Year and added this term to their dictionary’s database. In 2014 Google reported that its Android devices took 93 million selfies per day. The habit of regularly sharing one’s photographs on social media is usually associated with the inclusion of modern technologies in a person’s daily activities. But with the broad availability of smartphones and similar devices, some young people are addicted to posting selfies, while others post their photos from time to time, and some respondents do not do so at all.

According to lay psychology, there are various (and sometimes contradictory) stereotypes about excessive selfie-taking. It has been associated with self-centered-ness, or, vice versa, with the lack of self-confidence; with a desire for self-affirmation; or even with an excess of free time; with adherence to fashion, etc. However, once carefully studied, these assumptions are not always confirmed. Both positive and negative self-esteem can encourage users to engage in online social networking. Y. Wang and colleagues showed that selfie-posting was positively related to Chinese young adult women’s self-esteem, with positive feedback mediating this relationship (Wang et al., 2020). A study of adult men between the ages of 18 and 50 demonstrated an association between Instagram use, selfie manipulation, and body dissatisfaction (Modica, 2020). But S. Schebetenko (2019) did not find any correlation between self-esteem and the number of posts and portraits.

So, we are left with a lot of interconnected questions. Here are some of them:

1. Who uploads their photos to social networks more or less regularly?
2. What is the relationship between the characteristics of users and the features of the photos they upload?
3. What are the main motives for publishing self-portraits in on-line media by people with different personal characteristics?
4. Is there any correlation between the information that the user puts into his image and the information that the observer reads?
5. What are the psychological consequences of users’ activity on social networks?

Many studies show that respondents’ on-line behavior is associated both with their gender and age, as well as with personal characteristics such as, for example, the traits of the “Dark Triad:” narcissism (Boursier, Gioia, & Griffiths, 2020; Kim et al., 2016; Lee & Sung, 2016; Sorokowski et al, 2015); Machiavellianism; and psychopathy (Charoensukmongkol, 2016; Fox & Rooney, 2015). The differences in motivation and in quality and quantity of published selfies between women and men (Stefanone, Lackaff, & Rosen, 2011), as well as between younger and older respondents (Boursier & Manna, 2018; Dhir, 2016), are confirmed in most studies. Social media statistics also indicate that posting their own photos is most common among teens and young people.

Some studies have found more complex multifactorial relationships. For example, for male respondents, significant positive correlations were found between the traits of the “Dark Triad” (narcissism, Machiavellianism, and psychopathy) and the number of photos posted on the web, as well as the frequency of using photo editors to improve their appearance. The data on the role of narcissism in women are less clear (Fox & Rooney, 2015). It has been shown that people whose self-esteem is more based on approval from others are more likely to emphasize their appearance in online interactions by sharing more photos of themselves on Facebook (Stefanone et al., 2011). L. Monacis, M.D. Griffiths, and their colleagues (Monacis et al., 2020) demonstrated the mediating role of selfie behavior in the relationship of narcissism and psychopathy with social media addiction.

Personality traits affect the content of images posted in social media. For example, agreeable and conscientious users display more positive emotions in their profile pictures, while users high in openness prefer more aesthetic photos. Extraverts have a small face ratio, perhaps related to the multiple people present in their pictures, or the fact that they show more of their bodies or environment. Their photos are also strongly associated with not displaying reading glasses. Neuroticism is negatively correlated with colorfulness and display of emotions (Liu et al., 2016).

Women are more likely to publish black and white self-portraits, thus presenting themselves as art objects, and at the same time reducing the visibility of some textural defects of their skin. Men prefer the predominantly natural setting for selfies, while nearly 40% of female photographs are staged (Netusova, 2015).

Information “laid down by the sender” is not always adequately read by the “recipients.” This topic, first taken up in the works of A.A. Bodalev, has been developed in publications of recent years. V.L. Korinchuk and M.A. Shchukina showed that, when evaluating photographs which counseling psychologists posted on the portal for psychological services, potential clients and the participating psychologists themselves evaluated the photographs differently on such grounds as “everyday life,” “modesty,” “ordinary,” “realism,” “secrecy,” and whether the image was perceived as more empathic, professional, or expert (Korinchuk & Shchukina, 2019). Interesting data was also obtained in the study of the relationship between the personal characteristics of Instagram users and assessments of their virtual social status, made by experts unfamiliar with the individuals profiled (Belinskaya & Prilutskaya, 2019). Based on the profiles of respondents with a high level of neuroticism, Machiavellianism, narcissism, and self-monitoring, the experts quite consistently attributed to them a large number and quality of social connections and a high socio-psychological status.

Thus, the image chosen by a user of a social network is associated with her/his personal characteristics. At the same time, there are some data on the influence of the virtual image on human behavior in real life, the so-called Proteus effect (Reinhard et al., 2020; Yee & Bailenson, 2007).

In motivation research, subjects are most oft en asked to express their agreement/ disagreement with a set of statements about the reasons why they take selfies (for example, Sung et al., 2016), or an emphasis is placed on certain features of their behavior, as in the Selfitis Behavior Scale (Balakrishnan & Griffiths, 2018). On the basis of the theoretical models, the authors proposed questionnaires with a different number of factors (from 2 to 7). Despite the limitations of these techniques due to the culturally specificity of some questions, and the fact that they can elicit socially desirable responses, these studies gave very interesting results.

Research has confirmed that the main reasons that encourage people to take their photos and post them on social networks include the following: the desire to increase their self-esteem; communication; transfer and preservation of information; entertainment (Sung et al., 2016); or seeking self-approval, maintaining a sense of belonging, and preserving one’s memories and experiences (Etgar & Amichai-Hamberger, 2017). J. Balakrishnan and M.D. Griffiths (2018) propose a more differentiated approach to this topic by suggesting six factors of selfitis behavior: 1) self-confidence; 2) attention-seeking; 3) mood modification; 4) environmental enhancement; 5) subjective conformity; and 6) social competition.

In part, these motives coincide with traditional photography, and with the presentation and exchange of text information and pictures on social networks (for example, in LiveJournal), blogging, and other types of indirect communication. The main difference lies in the shift of the main focus to the image (figure, and more oft en to the face) of the author. In this case, the face or body which is “not evaluated,” *i.e.*, that has not received a large number of positive ratings (likes), is perceived as socially unsuccessful.

The struggle for likes underlies the creation of both a socially approved image, and its opposite – an image that “violates repressive norms of beauty” (Abeleva, 2014). E. Nguen (2014) showed that young girls and women (18-29 years old) post photos on Instagram mainly to get positive feedback. To achieve this goal, they actively use the various possibilities of lighting, choosing an angle and a scene, and more often experiment with new images. At the same time, girls tend to follow social norms more than boys. Perhaps this is due to the different attitudes of viewers towards female and male photographs: young men feel more freedom to show themselves without the risk of receiving disapproval, because their photos are criticized less often than girls’ ones (Burns, 2015). So, their network activity can be in part directed towards obtaining positive feedback to improve self-esteem.

The majority of the factors described are related to the subjective well-being of the person, although models of internal and external sides of the process of achieving well-being are still awaiting development (Perelygina, Rikel, & Dontsov, 2017). Our study does not pretend to solve this complex problem. We just wanted to study the relationship between the characteristics of respondents’ self-esteem and motivation (internal factors), and their perception of social support, social network behavior, and ideas about its causes (external factors), with their psychological well-being. So we posed as the main question: do people who are active in social networks and regularly post their selfies there, receive an additional resource for maintaining their self-esteem and psychological well-being from this activity?

The purpose of this preliminary study was to test the selfie motivation questionnaire and to examine the respondents’ ideas about the reasons they and their peers post selfies, and identify the differences, if any.

## Methods

### Participants

The pre-study sample included Russian undergraduate psychology students: N = 50 (36 women, 14 men; M _age_ = 22.17, SD_age_ = 4.22) who volunteered to take part in an off-line discussion about the role of selfies in their lives.

The main study sample was comprised of students (undergraduate and graduate level) from different universities in Moscow: N = 46 (all women; M_age_ = 26.96, SD-_age_ = 5.94).

### Measures

*Selfie motivation* was assessed by a selfie motivation questionnaire, which was composed of 15 items in 7-point Likert format scale (1= strongly disagree; 2 = disagree; 3 = somewhat disagree; 4 = neutral; 5 = somewhat agree; 6 = agree; 7 = strongly agree). Items were grouped into three subscales (five items in each subscale): 1) to maintain and increase self-confidence; 2) to maintain social contacts; and 3) to inform and preserve information. Thus, the minimum score on each subscale was 5, the maximum 35 (Nikitina, 2019; Nikitina, 2020).

To understand their *social network behavior,* we asked the participants about their avatars; the number of their on-line friends; time spent in Facebook/Instagram/ VKontakte; and the frequency of posting photos (including selfies) and checking their “Likes”.

We were interested in the following elements of *self-esteem*: dissatisfaction with one’s attractiveness and sociability, and the number of friends, calculated as the difference between ideal and real values of corresponding variables. *Motivation to success and to avoid failure* was studied by the T. Ehlers method.

The SOZU-22 questionnaire was used to assess *Emotional Support, Instrumental Support, Social Integration,* and *Satisfaction by Social Support,* while *Psychological Well-being* and its subscales (*Positive Relations, Autonomy, Management, Personal Growth, Aims in Life, and Self-Approval*) were measured by C. Ryff’s questionnaire.

### Procedure

The pre-study participants were engaged in oral discussion about the use of social networks, during which they were invited to fill in several forms. They indicated their age, gender, education, number of on-line and off-line friends, rated their own real and ideal attractiveness, and completed the selfie motivation questionnaire. They were also to assess their own agreement, and their assumptions about the agreement of their peers, with 15 statements regarding the reasons for doing selfies.

The respondents in the main study received a larger package of forms to fill out: the selfie motivation questionnaire; a questionnaire about their social media behavior; self-assessment scales of real and ideal attractiveness, sociability, and number of friends; C. Ryff’s scale of psychological well-being; the social support questionnaire SOZU-22; and T. Ehlers’ questionnaires on motivation for success and avoidance of failures.

Statistical analysis included the Mann-Whitney test for identifying intergroup differences, the Wilcoxon test for comparing the responses of respondents about their own motivations and the motivations of others, and Spearman’s correlation analysis. The results were considered significant when p<0.05.

## Results

During the pre-study, two participants indicated minimum scores for themselves and maximum scores for others concerning selfie motivation, and called the whole phenomenon “stupidity” and “mental illness.” These two respondents (both males) had no accounts on social networks; they also had the lowest number of off-line friends (0 and 1) and the greatest discrepancy between real and ideal attractiveness. Their results were excluded from further statistical analysis, but we will discuss them further later on in this paper.

The answers of 48 respondents (36 women, 12 men) confirmed that the main motives of young men and women were to increase self-esteem, maintain social contacts, and preserve and exchange information. The Wilcoxon test confirmed significant differences between the students’ ideas about their own and others’ reasons for selfie publication (see *Table 1*).

**Table 1:** Mean values of the Selfie motivation subscales

	Myself	Others	p
Self Confidence	**20.42**	**26.44**	**0.000**
Social Contacts	**9.94**	**15.34**	**0.000**
Information	21.90	23.92	0.068

*Note. Significant differences between the groups are in bold.*

The respondents indicated that they post photos mainly for themselves, using social networks as an archive. For example, the item “It’s faster than describing in words where, when, and with whom I was” got more agreement from the participants themselves than for the others (p = 0.000007). For their peers the students attributed the motivation of attracting the interest of others with the purpose of improving self-confidence. The highest agreement with the statement “If it will be impossible to get likes, then why upload photos!” was attributed to the others (p = 0.000000004).

Our participants believed that others are more motivated to post selfies, than they themselves. No significant differences in the responses of men and women, as well as in the answers of the respondents with different levels of network activity, were found. Twenty-five people (22 women, 3 men) gave their consent to the analysis of their real profiles on social networks. Young people less confident of their attractiveness were less likely to use portraits as avatars, and more often used external attributes in portraits (p < 0.05).

The next step was to examine whether the use of social media could be a resource for maintaining satisfactory self-esteem and psychological well-being.

First of all, we conducted an analysis of selfie motivation and psychological wellbeing. Only the idea that the selfie is a tool for storage and exchange of information correlated with well-being (Spearman Rho = 0.361, p < 0.01).

Then the participants in the main study (N = 46) were divided into two groups according to their activity in social networks and the number of their online “friends:” Group 1 (N1 = 20, mean age = 26.3) had fewer than 200 friends (average = 97.6); Group 2 (N2 = 26, mean age = 28.2) had more than 200 friends (average = 567.9). The group selection cutoff point, 200 friends, was chosen in accordance with Dunbar’s limit of a person’s stable social contacts (< 150–200 friends). When comparing the two groups’ results, we found significant differences (see *Table 2*).

**Table 2:** Mean values of the variables of the 2 groups of respondents

		Group 1 (<200 on-line friends)	Group 2 (>200 on-line friends)	p
Social networks behavior	Selfies per month	1.00	1.38	0.547
	Views of photo ratings, per month	4.60	27.75	0.003
	Off-line friends	**3.70**	**10.62**	**0.004**
Selfie motivation	To preserve and share information	20.30	24.38	0.007
	To show oneself and attract attention	19.00	21.69	0.286
	To get social support and meet group norms	11.30	13.92	0.284
Self-assessment	Attractiveness Real	69.63	63.31	0.350
	Sociability Real	53.50	64.38	0.078
	Many Friends Real	42.50	54.92	0.043
	Attractiveness Delta	14.00	19.08	0.119
	Sociability Delta	11.38	15.23	0.603
	Many Friends Delta	13.38	21.00	0.568
Social support (SOZU-22)	Emotional Support	35.60	38.77	0.283
	Instrumental Support	15.60	16.62	0.470
	Social Integration	26.60	28.46	0.154
	Satisfaction by Social Support	5.90	5.54	0.821
	Social Support (sum)	80.10	85.08	0.424
Psycholo gical well-being (C.Ryff )	Positive Relations	59.20	64.92	0.021
	Autonomy Management	56.50 55.70	56.67 59.58	0.670 0.220
	Personal Growth	61.70	69.42	0.000
	Aims In Life	62.40	72.00	0.000
	Self Approval	53.90	60.08	0.129
	CRyff Summ	**349.40**	**382.67**	**0.012**
Ehler’s motivation tests	Motivation to avoid failure	12.67	17.33	0.004
	Motivationfor success	17.00	16.78	0.733

*Note. Attractiveness, Sociability and Many Friends Delta indicators show the differences between the ideal and real values of the corresponding variables. Significant differences between the groups are in bold.*

At first glance the results of the respondents of Group 2 seemed to be more positive: they had more friends in real life, considered themselves more sociable, with an average of the same number of photos posted on the network, and they were several times more likely to view their “likes” (the first group had an average of 1 photo per month and 4.6 views, the second 1.38 and 27.75 views, respectively). On the scales of psychological well-being, Group 2 had significantly higher results in Positive relationships with others, Personal Growth, and Aims in Life. However, such a seemingly positive result was darkened by the data from the Ehlers tests; it was in Group 2 that the motivation to avoid failure scored higher (p < 0.01), and the motivation for success was somewhat lower. Those with many on-line friends also rated their attractiveness lower, and they had a greater discrepancy between real and ideal indicators. To clarify this situation, correlation analysis was performed separately for each group.

It turned out that in Group 2, almost all the scales of psychological well-being (except for autonomy) were associated with social support (p < 0.01), which in turn correlated with the number of friends in real life. Moreover, the more friends, the more social support, and the less pronounced motivation to avoid failure, the higher the indicators of well-being. But there was no effect of the number of on-line or offline friends on the motivation to avoid failure.

A different correlation structure was observed in the group of people with a more limited number of “friends” in social networks, most of whom were personal acquaintances (Group 1). These respondents did not show as many links between wellbeing and social support, and the discovered relationship between Satisfaction with Social Support and Personal Growth had a negative sign. At the same time, psychological well-being (Autonomy, Personal growth, and Aims in Life) in this group were associated with the motivation for success. Avoiding failure correlated only with the number of on-line friends (Spearman Rho = 0.754**) and number of off-line friends (Spearman Rho = -0.955**).

## Discussion

The first aim of this study was to uncover the motives of Russian respondents for publishing their selfies on social media. To avoid the social desirability effect in the answers, we asked the participants to answer the questions twice – once on behalf of themselves and once on behalf of their peers. Their own motivation was more internal, aimed at preservation of information, as well as maintaining contacts, while they attributed to others the desire to attract attention and follow group norms. The differences we found are consistent with the results of S. Diefenbach and L. Christoforakos (2017), who also showed that people more often attributed to others the motivation for self-presentation through selfies, while judging their own photographs to be more authentic. Nevertheless, the study participants confirmed the presence of all three groups of motives when posting selfies.

Those two subjects we mentioned earlier who did not admit the meaning of posting selfies by themselves, and believed that others were doing it foolishly, showed a small (1 and 0) number of friends in real life, and high values of dissatisfaction with themselves. Of course, the results of only two respondents are not enough to draw reliable conclusions, but it can be assumed that a complete rejection of online communication is not associated with its replacement with intensive offline interactions, and is not a good option. Some authors associate refusal to post their photos with lacking authenticity (Diefenbach, & Christoforakos, 2017)

The most important finding was that when separating the two groups of respondents on the basis of the level of their network activity, two different systems of connections between their senses of well-being with internal motivation and external support could be observed.

For Group 1, the well-being subscales were associated with the motivation for achieving success and only feeling instrumental support from others. These people rated their external attractiveness significantly higher, but sociability and number of friends lower, than those from Group 2. At the same time, the discrepancies between the real and ideal values of these characteristics were small, which showed the subjects’ satisfaction with their real state. At the same time, representatives of this group showed slightly lower indicators of well-being, not only on the scale of positive relationships with others, but also on the scale of Personal Growth and Aims in Life.

These last two scales included several items related to accepting the course of one’s life, while critical assessments were considered with a negative sign. In addition, these items can correlate positively with a desire for change and motivation for success.

The overall psychological well-being in Group 2 was higher. These respondents were more involved in their social environment, and they had many friends, but they would have liked to have even more, and to be more sociable. Their perception of social support was also somewhat higher, and it could be the main resource of their sense of wellbeing, as all the subscales of these two methods were closely related. At the same time, we observed a positive relationship between the failure avoidance motivation and feelings of social support, and a negative relationship between this motivation and well-being subscales. Perhaps in an effort to avoid failure, these respondents were getting used to relying on external support from the others. So for these groups, social activity in real life and close contacts in the virtual world may actually become a resource for well-being.

When continuing the study, it will make sense to take into account both the respondents’ marital status and their personal characteristics, especially the locus of control.

## Conclusion

In this study we found significant differences between the characteristics of women with high and low activity, and large and small number of “friends,” on social networks. Those for whom psychological well-being was largely determined by interaction with others often sought to avoid failure, so they included more contacts in their network, and more often looked at the responses to their photos. For these persons, communication (including virtual contacts) was the important resource of well-being. Representatives of the first group were less dependent on others, and assessed their appearance more positively; their motivation was directed to achieving success. For them psychological well-being was not related to their activity in social networks.

## Limitations

The first limitation of the study was its limited sample (its size, the absence of men’s data, and the fact that most of the sample were psychology students). The data collected were analyzed only quantitatively. We did not have enough measurements aimed at studying such personal characteristics of respondents as their locus of control and consciousness, to attain a deep understanding of the phenomenon.
